# Supplementary material for: A Proteomic Study of the Effect of N-acetylcysteine on the Regulation of Early Pregnancy in Goats
Source: Animals (Basel). 2022 Sep 15;12(18):2439. doi: 10.3390/ani12182439 (PMC9495164; doi:10.3390/ani12182439)
Supplement: Supplementary file 1 [file animals-12-02439-s001.zip › Supplementary.pdf]

Supplemental Table S1 Compositions and nutrients levels of the basal diets (air-dry basis)

raw materials	Percentage（%）	Nutritional level	Percentage（%）
Corn silage	50.00	DM	60.13
Chinese wildrye	20.00	Metabolic energy（MJ/kg DM）	12.51
Corn	15.00	Crude protein	13.42
Soybean meal	8.02	Organic matter	86.38
Wheat bran	4.98	NDF	38.67
Calcium bicarbonate	0.50	ADF	31.07
Sodium chloride	0.50	Ca	0.68
**Premix**[#]	1.00	P	0.49
Total	100.00		

[#]Per kilogram of premix of the diet contains vitamin A 55,000 IU, vitamin D 11,500 IU, vitamin E 13,000 IU, $MgSO_4·H_2O$ 110 g, $CuSO_4·5H_2O$ 0.7 g, $FeSO_4·7H_2O$ 3.0 g, $MnSO_4·H_2O$ 2.5 g, $ZnSO_4·H_2O$ 5.0 g, Na_2SeO_3 15 mg, KI 40 mg, $CoCl_2.6H_2O$ 28 mg. DM, dry matter; NDF, neutral detergent fibre; ADF, acid detergent fibre.

Supplemental Table S2 primer parameters of each gene

Gene name	Primer sequence（5'-3'）	GenBank ID	Fragment size /bp	Tm/℃
PELO	F：AACCGCCAGTTCACCCT<br>R：GCCTTTCCGTTTCCGAG	XM_005694722.2	201	57
SLC27A4	F：GCAATGAGTTTGTGGGT<br>R：CAGAAGAGGTTGAGCGA	XM_018055876.1	199	57
SLC2A1	F：TGATTGGTTCCTTCTCCG<br>R：AGGACTTGCCCAGTTTTG	NM_001314223.1	126	57
ITGAL	F：TTTGCGGCTGTTCAGTTTT<br>R：TTCTGTTCGTCGGTGGCTT	NM_001314316.1	239	57
GFAP	F：CAGTGGCGTCCAGCAACAT<br>R：CTCCAGGTCGCAAGTCAAG	XM_018065254.1	163	57
OLFML3	F：CAGAGCAGCAGTGGGATA<br>R：CGGGGGAAATAAGGGAGT	XM_005677850.3	178	57
TUBA4A	F：TGATGAGATCCGAAATGG<br>R：TAGTCAACAGAGAGCCGC	XM_018059034.1	258	57
TOMM20	F：AGCAAACTCTCCCACCACC<br>R：TCCACATCATCTTCAGCCA	XM_018042526.1	103	57
β-actin	F：AGATGTGGATCAGCAAGCAG<br>R：CCAATCTCATCTCGTTTTCTG	XM_018039831.1	139	57

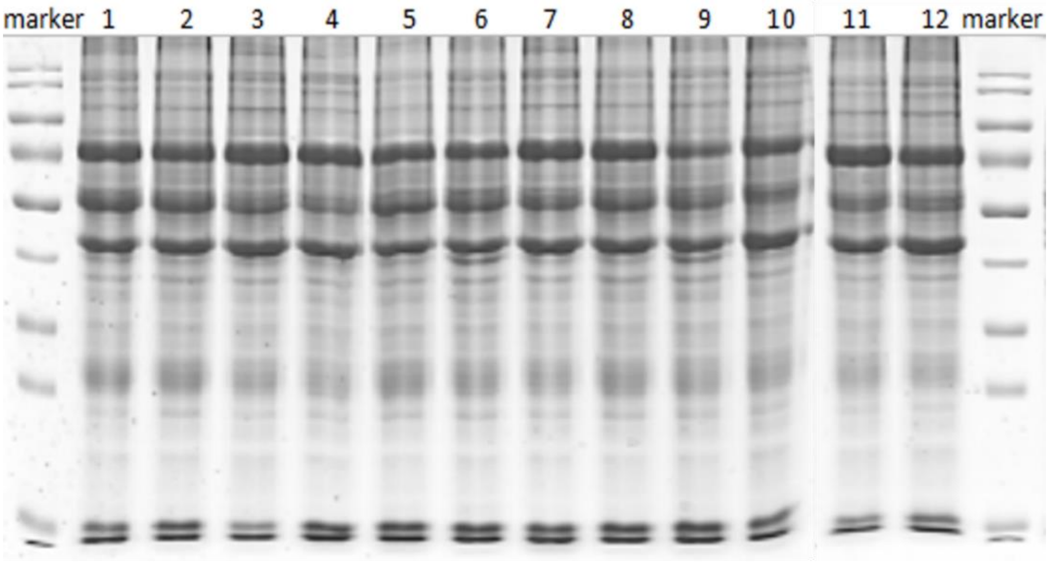

Supplemental Figure S1 Identification of uterine horn protein by SDS-PAGE

SDS-PAGE gel electrophoresis to test the quality of ewe uterine keratin samples

The loading amount is 15ug, which 1-6 is the control group, and 7-12 is the N-acetylcysteine group

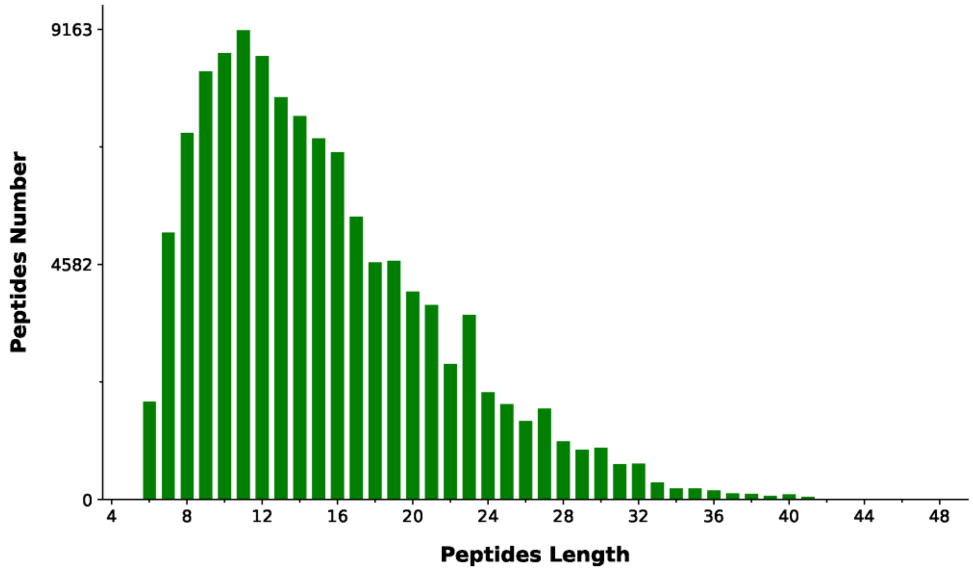

Supplemental Figure S2 Peptide length distribution

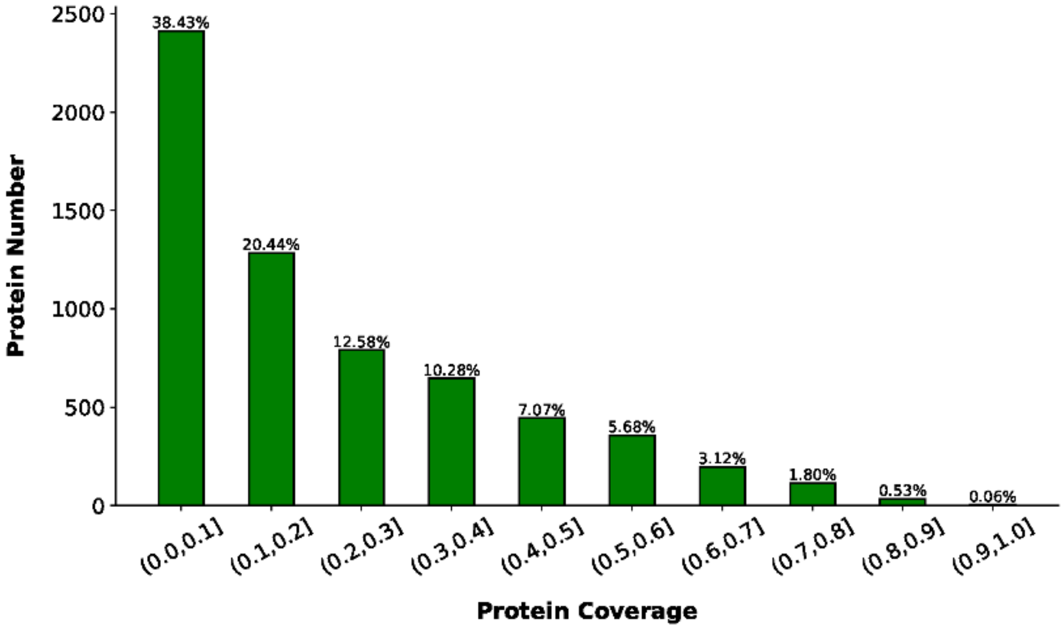

Supplemental Figure S3 Protein coverage distribution

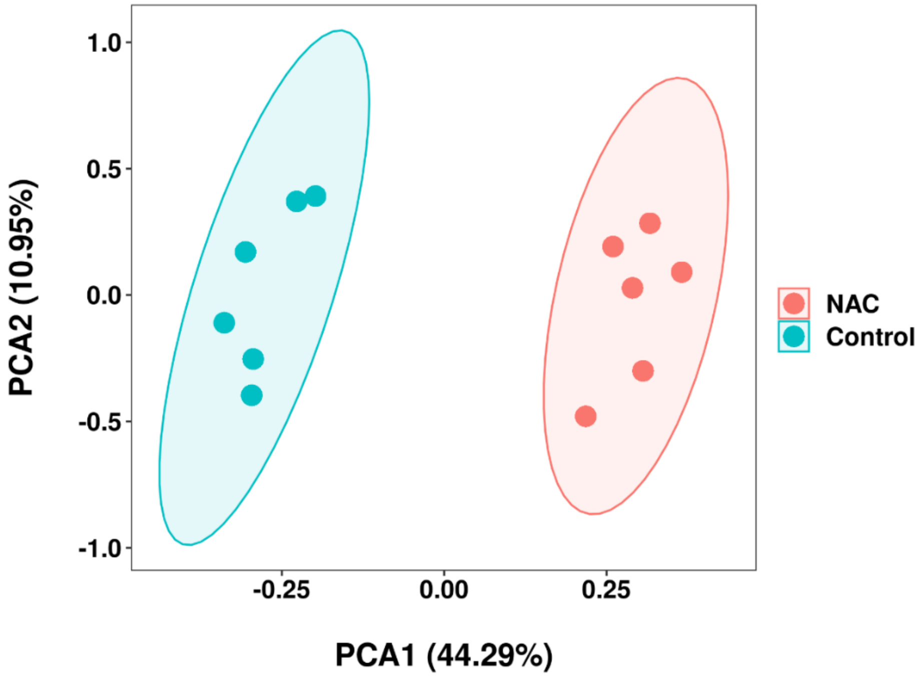

Supplemental Figure S4 Principal component analysis

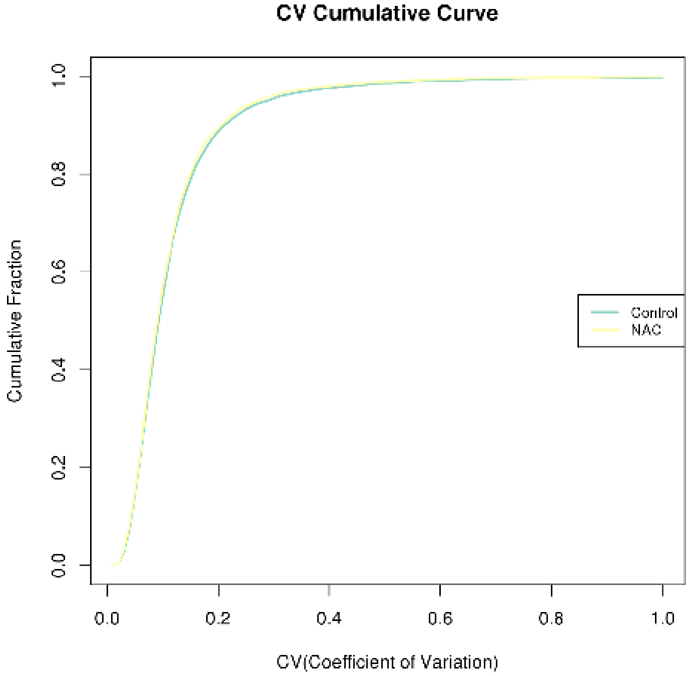

Supplemental Figure S5 Coefficient of variation analysis
